# Clinical significance and mechanistic role of Hsa_circ_0005075 in recurrent spontaneous abortion: Regulation of trophoblast proliferation and invasion through the Wnt/β-catenin pathway

**DOI:** 10.1016/j.clinsp.2025.100816

**Published:** 2026-02-13

**Authors:** Meng Li, Shuyuan Liu, Xuan Du

**Affiliations:** a Department of Family Planning, Weifang People's Hospital, Weifang, Shandong, China; b Department of Reproductive Health, W.F. Maternal and Child Health Hospital, Weifang, Shandong, China; c Chronic Disease Service Center, Weifang People's Hospital, Weifang, Shandong, China

**Keywords:** Hsa_circ_0005075, Recurrent spontaneous abortion, Biomarker, Trophoblast, Invasion, Apoptosis, Wnt/β-catenin pathway, Cell cycle, circRNA

## Abstract

**Objective:**

Given the current lack of diagnostic markers with high sensitivity and specificity, the diagnosis and treatment of Recurrent Spontaneous Abortion (RSA) have always presented significant difficulties. To analyze the relationship between hsa_circ_0005075 and RSA and conduct a preliminary exploration of its mechanism.

**Methods:**

Fifty-eight RSA patients admitted to the studied hospital from March 2022 to January 2024 (research group) and 52 normal pregnant women (control group) were selected as the research subjects. Peripheral blood hsa_circ_0005075 expression was detected to determine the difference between the two groups. In addition, the evaluation value of hsa_circ_0005075 for RSA, miscarriage, and adverse pregnancy outcomes was analyzed through Receiver Operating Characteristic (ROC) curves. Furthermore, human placental Trophoblast Cells (TBCs) HTR8/SVneowere purchased and transfected with abnormal hsa_circ_0005075 expression sequences to identify changes in HTR8/SVneoproliferation, invasion, and apoptosis, and alterations in Wnt/β-catenin pathway expression.

**Results:**

hsa_circ_0005075 was higher in the research group than in the control group (4.00 ± 0.93 vs. 2.78 ± 0.94, *p* < 0.05). ROC curves indicated the excellent diagnostic effect of hsa_circ_0005075 on the occurrence of RSA (Area Under Curve [AUC = 0.820]), miscarriage (AUC = 0.744), and adverse pregnancy outcomes (AUC = 0.726). In vitro, increasing hsa_circ_0005075 inhibited HTR8/SVneogrowth, decreased cell clones and cell invasion number, increased apoptosis, shortened the G0/G1 phase, and activated the Wnt/β-catenin pathway; the opposite results were observed after silencing hsa_circ_0005075 expression. Mechanistically, gain and loss-of-function experiments revealed that hsa_circ_0005075 acts as a negative regulator of trophoblast proliferation, invasion, and cell cycle progression, and induces apoptosis by suppressing the Wnt/β-catenin signaling pathway.

**Conclusions:**

The present findings identify hsa_circ_0005075 as a promising circulating biomarker for RSA and demonstrate that it contributes to RSA pathogenesis by impairing trophoblast function through inhibition of the Wnt/β-catenin pathway, suggesting its potential as a therapeutic target.

## Highlights

• Serum hsa_circ_0005075 is significantly elevated in patients with Recurrent Spontaneous Abortion (RSA).
• Hsa_circ_0005075 exhibits high diagnostic value (AUC = 0.820) for distinguishing RSA patients from healthy controls.
• Overexpression of hsa_circ_0005075 suppresses trophoblast cell proliferation, invasion, and cell cycle progression while promoting apoptosis.
• Hsa_circ_0005075 exerts its functional effects by inhibiting the activation of the Wnt/β-catenin signaling pathway.
• Targeting hsa_circ_0005075 may represent a novel diagnostic and therapeutic strategy for RSA.

## Introduction

Recurrent Spontaneous Abortion (RSA) refers to three or more consecutive miscarriages before the 28th week of pregnancy, with complicated etiology and lack of specific manifestations.^1^ Statistics show that RSA, with an increasing trend in recent years, is found in approximately 1 %‒5 % of all pregnant women globally.^2^ Clinically, it is believed that many factors, such as chromosomal, endocrine, and immune dysfunction, may lead to RSA. Given the current lack of diagnostic markers with high sensitivity and specificity, the diagnosis and treatment of RSA have always presented significant difficulties.^3^ With the deepening of research, molecular pathogenesis has gradually become a new direction for the diagnosis and treatment of various diseases. For RSA, researchers have also begun to search for potential diagnosis and treatment indicators from this perspective.

Circular RNAs are a class of endogenously covalently bonded single-stranded RNAs that function through miRNA sponges and regulation of gene expression.^4^ At present, studies have confirmed that circular RNAs have a close relationship with pregnancy diseases such as eclampsia and gestational diabetes^5^^,^^6^ and their role in RSA has begun to attract much attention. Hsa_circ_0005075 is a newly discovered member of the circ_cRNA family, which is highly expressed in colorectal cancer and promotes tumor cell proliferation and invasion.^7^ In a study on the effect of hsa_circ_0005075 on drug-resistant ovarian cancer cells by Wang YM et al., hsa_circ_0005075 was proposed to have a direct relationship with the function of female ovaries and greatly affects the normal activity of ovarian cells.^8^

These studies suggest that hsa_circ_0005075 is also potentially related to the progression of pregnancy and may be involved in the occurrence and development of RSA. However, there is no research that confirms the specific relationship between hsa_circ_0005075 and RSA, and the authors are unable to determine the exact impact of hsa_circ_0005075 on RSA. Therefore, this study will explore the clinical significance of hsa_circ_0005075 in RSA and conduct a preliminary exploration of its mechanism of action, thereby providing new reference opinions for the diagnosis and treatment of RSA in the future.

## Information and methodology

### Study population

The confidence interval was set to 95 % CI, statistic (*Z* = 1.96), Error (*E* = 10 %), Probability (*P* = 0.5), and the sample size (*N* = 96) was obtained according to the formula N=Z2×[P×(1−P)]/E2. With this as the minimum requirement, the sample was taken from the patients with RSA who were admitted to the hospital in the period of March 2022 to January 2024. Finally, 104 patients with RSA were enrolled in this study (research group), and in a ratio of 1:1, 104 normal pregnant women who visited during the same period were selected as the control group. This study was conducted in strict adherence to the STROBE statement, and for diagnostic and prognostic analyses, the authors will also strictly follow the STARD guidelines. In addition, the Ethics Committee of the studied institution approved this study (WFYXY-2024–19), and all study subjects signed an informed consent form.

### Inclusion-exclusion criteria

Inclusion criteria for the research group: Age > 18-years-old; meeting the RSA diagnostic criteria^9^ (normal karyotypes of both the husband and wife, normal development of the reproductive system, and at least 3 consecutive spontaneous miscarriages in early pregnancy); no use of anticoagulant therapy or fibrinolytic drugs, hormones, or immunosuppressants within the recent two months. Patients with spontaneous abortion for other reasons, diabetes, cardiovascular diseases, or immune system diseases, as well as those with incomplete clinical data, were excluded. Inclusion criteria for the control group: Confirmed to be in the early stage of pregnancy by B-ultrasound and Human Chorionic Gonadotropin (HCG) test, with no history of spontaneous abortion nor systemic diseases such as diabetes and autoimmune diseases.

### Sample collection and testing

Fasting venous blood (3 mL) was collected from both groups upon admission into coagulation-promoting tubes, and the serum was separated by centrifugation (3000 rpm/min) for 10 min after standing at room temperature for 30 min. Total RNA in serum was extracted with a Plasma Midi Kit and verified for its concentration, after which the cDNA was obtained by reverse transcription according to the instructions of Hifair III 1st Strand cDNA Synthesis Super Mix for qPCR. Guangzhou Geneseed Biotech Co., Ltd. was entrusted to design the primer sequences (Table 1) for PCR. Reaction conditions: 95 °C for 2 min, 95 °C for 10 s, and annealing/extension at 60 °C for 30 s, for 40 cycles. hsa_circ_0005075 expression relative to 18S was calculated by 2^−△△Ct^.

**Table 1:** Sequence of primers.

	**F (5′−3′)**	**R (5′−3′)**
**hsa_circ_0005075**	CCCCTGGACTCTCTCAAAATTC	GTCTTGCCACTTGTGTTACCG
**18S**	AGGGACAAGTGGCGTTCAGC	CGGACATCTAAGGGCATCAC

### Follow-up investigation

A pregnancy follow-up investigation was conducted on the patients in the research group. The survey items included whether the pregnancy was completed and whether the patients who completed the pregnancy had adverse pregnancy outcomes. Adverse pregnancy outcomes were defined as the presence of difficult labor, premature birth, stillbirth, neonatal developmental malformations, or congenital diseases.

### Cellular data

Human placental Trophoblast Cells (TBCs) HTR8/SVneowere purchased from the American Type Culture Collection (ATCC) and cultured in a medium containing 10 % fetal bovine serum, 100 μg/mL streptomycin, and 100 μg/mL penicillin. Passage was performed every 2‒3 days, and cells in the logarithmic phase were taken for subsequent experiments.

### Cell transfection

The mimic (Mimics), inhibitor (Inhibitors), and negative control (nc) sequences of hsa_circ_0005075 were transfected into HTR8/SVneofollowing the Lipofectamine™ 2000 kit instructions, and the transfection success rate was confirmed by PCR detection of hsa_circ_0005075 expression.

### Cell activity detection

Cells were seeded onto the wells of a 96-well plate with four replicate wells set up in each group. CCK-8 solution with a volume of 10 μL was added to one well at 0-, 24-, 48-, and 72-hours of culture, and Dimethylsulfoxide (DMSO) was added to terminate the growth after 2-hours of culture. A microplate reader was used to determine the absorbance value at 450 nm, and the cell growth curve was plotted. Cells were plated in 6-well plates with 500 cells/well, and 3 replicate wells were set for each group. Following medium removal after 2-weeks of cultivation, the cells were formaldehyde-immobilized for 15-minutes and Giemsa-stained for 20-minutes. Clones with >50 cells were counted. Cloning rate = number of clone formations/number of cells inoculated × 100 %.

### Cell invasion detection

The logarithmic growth phase cells were suspended in serum-free DMEM to make a single-cell suspension (5 × 10^5^/mL). 0.25 mL of the sample was then inoculated on the Transwell upper chamber that was pre-treated with matrigel for 5-hours. 600 μL of 10 % fetal bovine serum containing DMEM was placed into the lower chamber. After 24 h of culture, the transmembrane cells were collected, fixed with 4 % paraformaldehyde, and stained with crystal violet for subsequent observation of the number of invading cells under the microscope.

### Apoptosis detection

After digestion, the cells in each group were gathered for resuspension to a 1 × 10^6^/mL solution, followed by PBS cleaning and 5 min of centrifugation (800 rpm/min; repeated twice) to discard the supernatant. 5 μL each of Annexin V-FITC and PI reagents were then added for 15 min of room temperature incubation, after which flow cytometry was utilized for apoptosis rate and cell cycle detection.

### Wnt/β-catenin pathway protein measurement

After tissue lysis by RIPA, 2.0 mL of the sample was centrifuged (10,000 r/min, 30 min) to extract the supernatant, and the protein concentration was determined. Following solution preparation according to the sample, Sodium Dodecyl Sulfate-Polyacrylamide Gel Electrophoresis (SDS-PAGE) was carried out. The liquid required for a 50 μg protein-containing sample was calculated, the sample was loaded and electrophoresed, and the gel running was terminated when the Marker protein was placed at the bottom of the glass plate. The Polyvinylidene Fluoride (PVDF) membrane was removed from the Trans-Blot instrument to be rinsed with Tris-Buffered Saline with Tween (TBST) for 5 min and immersed in diluted Wnt1 (1:1000), β-catenin (1:1000), and GAPDH (1:1000) primary antibodies. The next day, a horseradish peroxidase-labeled secondary antibody (1:2000) was added for a 2-hour culture. Following the addition of a developer to the PVDF membrane in a dark environment, imaging and analysis were performed using the Image-Lab system.

### Endpoints

**Fig. 1:**
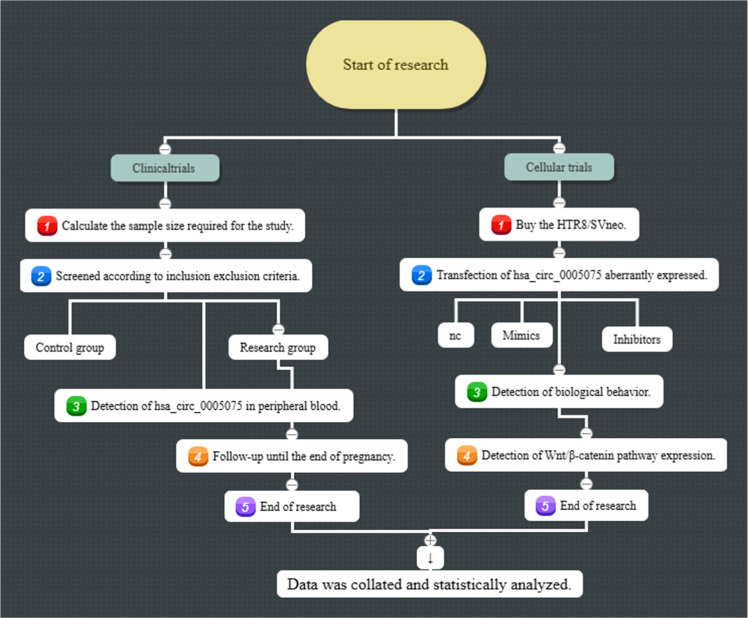
Flow of this study.

The differences in hsa_circ_0005075 expression between the research group and the control group were analyzed, and the value of hsa_circ_0005075 in assessing RSA, miscarriage, and adverse pregnancy outcomes was explored. In addition, the biological behavior of HTR8/SVneoand the Wnt/β-catenin pathway expression were observed after interfering with hsa_circ_0005075 expression. Fig. 1 illustrates the flow of this study.

### Statistical methods

Statistical analysis was performed using SPSS 24.0 software. The comparison of count data [n ( %)] was performed using the Chi-Square test; the inter-group comparison of measurement data (*χ*±*s*) used the independent sample *t*-test, and the multi-group comparison employed the repeated-measures analysis of variance and LSD intra-group test. The diagnostic value was analyzed using the Receiver Operating Characteristic (ROC) curves. Statistical significance is reported at the *p* < 0.05 level.

## Results

### Comparison of clinical data

Comparing the age, smoking, alcohol consumption, and other data between the research and control groups, the authors found no statistically significant difference (*p* > 0.05), indicating comparability (Table 2).

**Table 2:** Comparison of clinical data.

**Groups** **(*n*=104)**	**Age**	**Gestational week at study entry**	**Number of abortions (induced + uninduced)**	**History of childbearing**	**Smoking**	**Drinking**	**Final pregnancy outcome**
				**Yes/No**	**Yes/No**	**Yes/No**	**Miscarriage / No miscarriage**
Control	30.29 ± 2.92	8.28 ± 2.07	0.33 ± 0.49	9 (8.65) / 95 (91.35)	22 (21.15) / 82 (78.85)	14 (13.46) / 90 (86.54)	3 (2.88) / 101 (97.12)
Research	31.07 ± 3.70	8.12 ± 1.92	2.63 ± 0.65	3 (2.88) / 101 (97.12)	30 (28.85) / 74 (71.15)	15 (14.42) / 89 (85.58)	40 (38.46) / 64 (61.54)
*t* (χ^2^)	1.687	0.591	29.020	3.184	1.621	0.040	40.130
P	0.093	0.555	<0.001	0.074	0.203	0.841	<0.001

### Clinical expression of hsa_circ_0005075

**Fig. 2:**
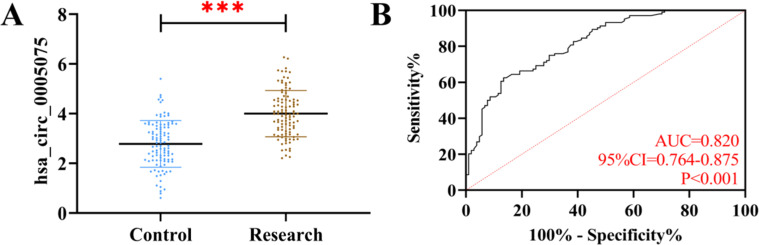
**Clinical expression of hsa_circ_0005075.** (A) Comparison of hsa_circ_0005075 expression between study and control groups. (B) ROC curves for hsa_circ_0005075 diagnosis of RSA occurrence (***p < 0.001).

The serum hsa_circ_0005075 in the research group was detected to be (4.00 ± 0.93), which was significantly elevated compared to the control group (*p* < 0.05). ROC curve analysis showed that when hsa_circ_0005075 > 3.66, its sensitivity and specificity for diagnosing RSA were 62.50 % and 86.54 %, respectively (*p* < 0.05) (Fig. 2).

### Clinical significance of hsa_circ_0005075 in RSA

**Fig. 3:**
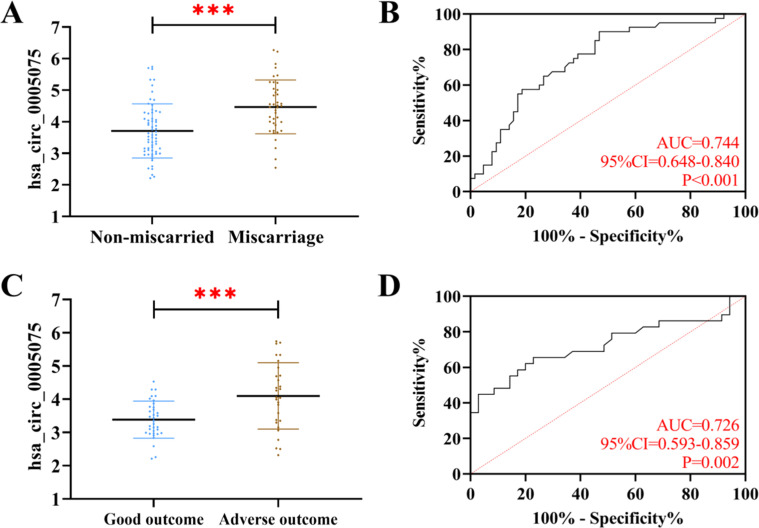
**Clinical significance of hsa_circ_0005075 in RSA.** (A) Comparison of hsa_circ_0005075 between miscarriage and non-miscarriage patients. (B) ROC curves for hsa_circ_0005075 diagnosis of miscarriage in RSA patients. (C) Comparison of hsa_circ_0005075 between adverse pregnancy outcomes and good pregnancy outcomes patients. (D) ROC curve of hsa_circ_0005075 for diagnosis of adverse pregnancy outcome (*** *p* < 0.001).

The follow-up showed that 64 patients in the research group did not have a miscarriage. The patients who miscarried showed evidently higher hsa_circ_0005075 than those without miscarriage (*p* < 0.05). When hsa_circ_0005075 > 3.64, its sensitivity and specificity for diagnosing miscarriage in RSA patients were 90.00 % and 53.13 %, respectively (*p* < 0.05). Among the non-miscarried patients, 29 cases had adverse pregnancy outcomes; by comparison, the hsa_circ_0005075 of patients with adverse pregnancy outcomes was higher than that of patients without (*p* < 0.05). When hsa_circ_0005075 > 3.80, its sensitivity for diagnosing adverse pregnancy outcomes in non-miscarried RSA patients was 65.52 %, and the specificity was 77.14 % (*p* < 0.05) (Fig. 3).

### Influence of hsa_circ_0005075 on TBCs

**Fig. 4:**
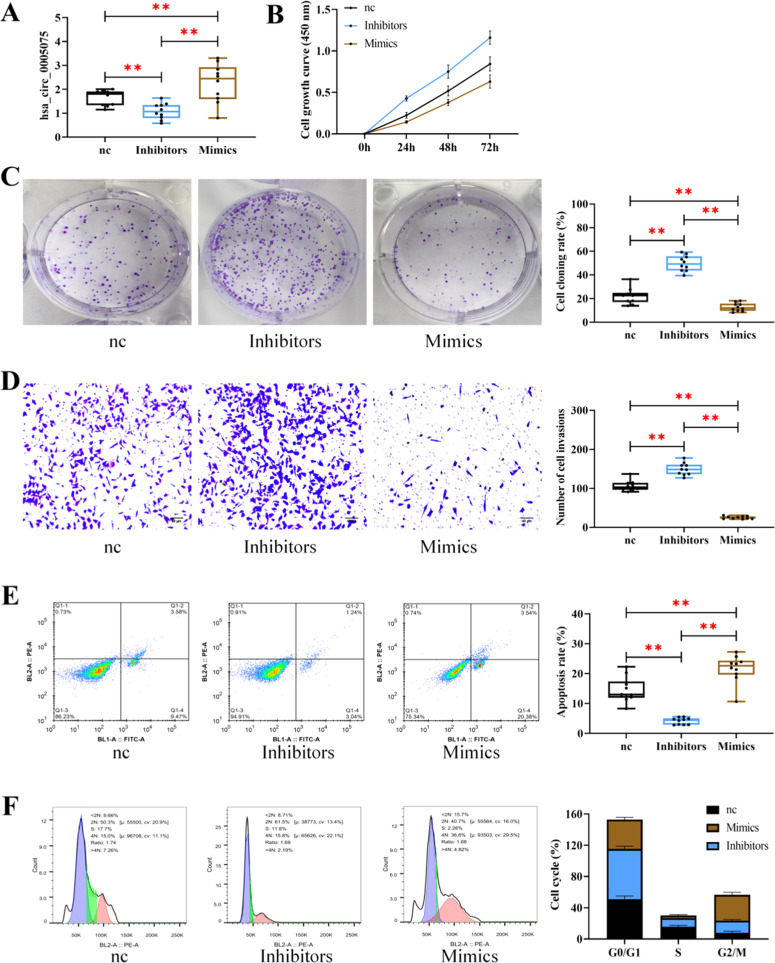
**Influence of hsa_circ_0005075 on TBCs.** (A) Expression of hsa_circ_0005075 in HTR8/SVneoafter transfection with an aberrantly expressed hsa_circ_0005075 sequence. (B) Effect of hsa_circ_0005075 on growth capacity. (C) Effect of hsa_circ_0005075 on the cloning ability of HTR8/SVneo. (D) Effect of hsa_circ_0005075 on HTR8/SVneoinvasion ability. (E) Effect of hsa_circ_0005075 on HTR8/SVneoapoptosis rate. (F) Effect of hsa_circ_0005075 on HTR8/SVneocell cycle (** *p* < 0.01).

First, the expression of hsa_circ_0005075 in three groups of cells was detected. It can be seen that the expression was the highest in the Mimics group and the lowest in the Inhibitors group (*p* < 0.05), confirming the successful transfection. The biological behavior detection results showed that the cell growth ability, cloning rate, and invasion number were lower in the Mimics group than in the Inhibitors group and the nc group, and the apoptosis rate was higher (*p* > 0.05); while the Inhibitors group exhibited higher cell growth ability, clone rate, and invasion number as well as lower apoptosis than the nc group (*p* < 0.05). According to the cell cycle analysis, the G0/G1 phase was shorter in the Mimics group versus the nc group, while that in the Inhibitors group was longer compared to the nc group (*p* < 0.05) (Fig. 4).

### Effect of hsa_circ_0005075 on the wnt/β-catenin pathway

**Fig. 5:**
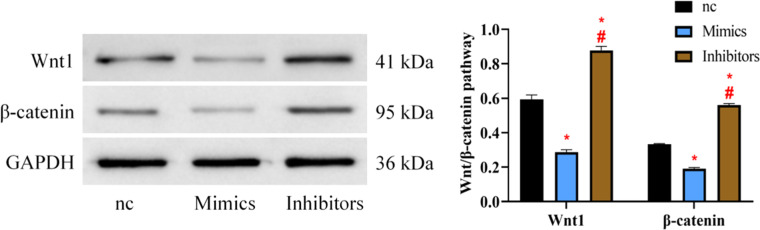
Effect of hsa_circ_0005075 on Wnt/β-catenin pathway protein expression in HTR8/SVneo. vs. nc group * *p* < 0.05, vs. Mimics group # *p* < 0.05.

Finally, the detection results of Wnt/β-catenin pathway expression showed that the Wnt1 and β-catenin protein expression of the Mimics group was reduced compared to the nc and Inhibitors groups, while that of the Inhibitors group was higher versus the nc group (*p* < 0.05) (Fig. 5).

## Discussion

In recent years, the incidence of RSA has been rising year by year, imposing a significant burden on patients, newborns, and their families. How to diagnose and treat RSA effectively is the key to ensuring the health of patients and newborns. In this study, the authors found that hsa_circ_0005075 and RSA were closely linked, providing a new reference for future clinical searches for clinical markers of RSA.

Hsa_circ_0005075 is located on chromosome 1:2137,735,821,415,706. Early studies mainly focused on its role in various tumor diseases, such as liver and colorectal cancers,^10^^,^^11^ but its relationship with RSA remained to be confirmed. In this study, the authors found significantly higher hsa_circ_0005075 in the research group compared to the control group, suggesting that hsa_circ_0005075 may be involved in the occurrence and development of RSA. Meanwhile, in previous studies, the authors also observed that hsa_circ_0005075 was upregulated in diseases such as neuropathic pain,^12^^,^^13^ which is consistent with the present results. Moreover, the authors observed aberrantly expressed hsa_circ_0005075 in RSA patients with or without miscarriage, as well as non-miscarried patients with or without adverse pregnancy outcomes, further supporting the relationship between hsa_circ_0005075 and RSA and laying a reliable foundation for hsa_circ_0005075 as a clinical evaluation index of RSA.

Through ROC curve analysis, the authors found that hsa_circ_0005075 showed excellent diagnostic effects on the occurrence of RSA, miscarriage, and adverse pregnancy outcomes, with AUCs reaching 0.820, 0.744, and 0.726, respectively, demonstrating high reference value. Currently, there are no effective clinical diagnostic indicators for RSA in clinical practice. Clinically, it is not only necessary to determine the recurrence of RSA through examinations, but also difficult to conduct a dynamic assessment of the disease's progression, which leads to significant limitations in the diagnosis and treatment of RSA.^14^ As one of the genetic materials in the human body, circ_cRNAs themselves have a relatively stable molecular structure and can be detected in samples such as blood, tissues, body fluids, and cells.^15^ There will be no human error in the results of quantitative analysis, which has many advantages such as objectivity, accuracy, and convenience. In this study, the excellent evaluation effect of hsa_circ_0005075 on the progression of RSA can not only achieve large-scale clinical screening, but also allow for timely adjustment of treatment intervention measures based on the test results to protect the health of patients. However, given that hsa_circ_0005075 has also been found to exhibit abnormal expression in diseases such as liver cancer,^16^ the authors believe that, in actual clinical applications, a comprehensive assessment should be conducted in combination with the symptoms of RSA or other examination results.

In the above text, the authors have preliminarily determined the relationship between hsa_circ_0005075 and RSA, but its mechanism of action remains unclear. TBCs are the main cells that make up the placenta, and their structural integrity and cellular activity largely determine the occurrence of miscarriage.^17^ Therefore, the authors transfected the abnormal expression sequences of hsa_circ_0005075 into HTR8/SVneoto observe changes in its activity. The results showed that after increasing the expression of hsa_circ_0005075, the cell growth ability, cloning rate, and invasion number of HTR8/SVneodecreased, the apoptosis rate increased, and the G0/G1 phase shortened, while silencing the expression of hsa_circ_0005075 resulted in completely opposite results. That is to say, highly expressed hsa_circ_0005075 can inhibit the activity of TBCs and promote their apoptosis, which is also the main mechanism by which hsa_circ_0005075 participates in RSA, validating the higher hsa_circ_0005075 expression in the research group compared to the control group and the higher expression in miscarriage patients than in non-miscarriage patients in the above text. In addition, the enhanced activity of HTR8/SVneoby silencing hsa_circ_0005075 also suggests that targeted silencing of hsa_circ_0005075 may be a new treatment method for RSA. However, it still needs a lot of experimental verification to realize its clinical application.

**Fig. 6:**
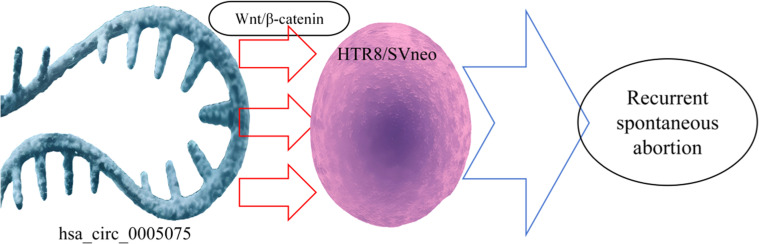
hsa_circ_0005075 regulates trophoblast involvement in RSA through the Wnt/β-catenin pathway.

Finally, when reviewing relevant studies, the authors found that the current hsa_circ_0005075 pathways mainly involve two pathways, namely the SIRT1 pathway and the Wnt/β-catenin pathway,^18^^,^^19^ among which the relationship between hsa_circ_0005075 and the Wnt/β-catenin pathway has attracted the authors’ attention. Zhou M et al. found that USP7 prevented spontaneous abortion in RSA patients through the Wnt/β-catenin pathway,^20^ while Li N et al. showed that downregulation of the Wnt/β-catenin pathway can cause TBC dysfunction.^21^ Therefore, combined with the above research results, the authors speculate that the influence of hsa_circ_0005075 on RSA may also be related to the Wnt/β-catenin pathway. In order to verify this view, the authors further tested the expression of the Wnt/β-catenin pathway. The expression of Wnt1 and β-catenin was found to be decreased in the Mimics group but increased in the Inhibitors group, indicating that highly expressed hsa_circ_0005075 also has the effect of inhibiting the Wnt/β-catenin pathway. These results also help us to further understand the mechanism by which hsa_circ_0005075 affects RSA. Fig. 6 demonstrates what the authors learned about the relationship between hsa_circ_0005075 and RSA based on the results of this study.

Of course, more in-depth analysis is needed to explore the influence of hsa_circ_0005075 on RSA in the future, such as finding its downstream targeted genes and the effects of targeted silencing of its expression on live animals. Besides, more cases should be included to further confirm the clinical significance of hsa_circ_0005075 in RSA. It is important to note that this study is only a simple analysis of the effect of hsa_circ_0005075 knockdown on the expression of the Wnt/β-catenin pathway, and these results cannot fully reveal the mechanism of action of hsa_circ_0005075 on the Wnt/β-catenin pathway. In the follow-up study, the authors also need to observe whether hsa_circ_0005075 has changed by counter-regulating the Wnt/β-catenin pathway, so as to help clinicians more deeply and comprehensively understand the relationship between hsa_circ_0005075 and the Wnt/β-catenin pathway.

## Conclusion

The present study provides compelling clinical and mechanistic evidence that hsa_circ_0005075 is a crucial player in RSA pathogenesis. The authors not only established its significant upregulation in RSA patients but also validated its strong diagnostic potential as a non-invasive circulating biomarker. Furthermore, the authors demonstrated that elevated hsa_circ_0005075 directly impairs key trophoblast functions ‒ including proliferation, invasion, and survival ‒ by attenuating Wnt/β-catenin pathway activity. These findings position hsa_circ_0005075 not only as a valuable clinical indicator but also reveal a novel molecular axis contributing to RSA. Future research focusing on therapeutic silencing of hsa_circ_0005075 in vivo and exploring its downstream targets holds promise for developing new intervention strategies for this challenging condition.

## Funding

The authors declare that no funds, grants, or other support were received during the preparation of this manuscript.

## Authors’ contributions

Xuan Du designed and supervised the study, Meng Li wrote and revised the manuscript, Shuyuan Liu collected and analyzed data, and Meng Li visualization of the data. All authors read and approved the final submitted manuscript.

### Ethical approval

The study protocol was approved by the Ethics Committee of Weifang People's Hospital (Approval number: WFYXY-2024–19).

### Consent to publish

All authors gave final approval of the version to be published.

### Data availability statement

The data used to support the findings of this study are available from the corresponding author upon request.

## Declaration of competing interest

The authors declare that they have no known competing financial interests or personal relationships that could have appeared to influence the work reported in this paper.
